# Stereotactic or Conventional Radiation for Early-Stage Non-small Cell Lung Cancer: A Systematic Review and Meta-Analysis

**DOI:** 10.7759/cureus.38198

**Published:** 2023-04-27

**Authors:** Adam Mutsaers, Tina Wanting Zhang, Alexander Louie, George Rodrigues, David Palma, Melody Qu

**Affiliations:** 1 Radiation Oncology, Sunnybrook Health Sciences Centre - Odette Cancer Centre, Toronto, CAN; 2 Radiation Oncology, British Columbia (BC) Cancer, Vancouver, CAN; 3 Radiation Oncology, Victoria Hospital, London Health Sciences Centre, London, CAN; 4 Medicine & Dentistry, Western University, London, CAN

**Keywords:** cfrt, systematic review and meta-analysis, early-stage lung cancer, conventional radiation, nsclc, sabr

## Abstract

Stereotactic ablative radiotherapy (SABR) has been increasingly used for the treatment of inoperable early-stage non-small cell lung cancer (NSCLC). It has been shown to provide promising local control (LC) and toxicity in prospective trials. However, randomized trials have shown conflicting results in terms of whether SABR confers an overall survival (OS) advantage compared to conventionally fractionated radiotherapy (CFRT). A systematic review of Medline and Embase (inception to December 2020) was performed on early-stage NSCLC patients randomized to SABR versus CFRT. Two independent reviewers screened titles, abstracts, and manuscripts. A random-effects model was used to estimate treatment effects. Toxicity outcomes were compared by the Cochran-Mantel-Haenszel test. Individual patient data were digitally approximated and pooled as secondary analysis. The literature search identified 1494 studies, and 16 studies were included for full-text review. Two randomized trials were identified, including a total of 203 patients, of which 115 (57%) received SABR, and 88 (43%) received CFRT. The weighted mean age was 74 years and 48% of patients were male. Most patients had T1 cancers (67%). Stereotactic ablative radiotherapy was not associated with a significant improvement in OS (hazard ratio: 0.84; 95% confidence interval (CI) 0.34-2.08, p=0.71). There was no significant difference in LC between SABR and CFRT (relative risk: 0.59; CI 0.28-1.23, p=0.16). Of the commonly reported adverse events, one grade 4 toxicity of dyspnea was reported for SABR, while all others i.e., grade 3 or higher toxicities were similar. Stereotactic ablative radiotherapy demonstrated less esophagitis, dyspnea, and skin reaction of any grade. Despite widespread adoption and extensive single-arm prospective and retrospective studies suggesting its benefit, this systematic review and meta-analysis of randomized trials fail to confirm improvements in LC, OS, and toxicity profile of SABR over CFRT in early NSCLC. This small study is likely underpowered to detect clinically significant differences.

## Introduction and background

Over the past two decades, stereotactic ablative radiotherapy (SABR) has been increasingly used for the treatment of inoperable early-stage non-small cell lung cancer (NSCLC)[1]. This has been driven by the pivotal single-arm Radiation Therapy Oncology Group (RTOG)-0236 trial demonstrating excellent local control (LC) rates with SABR, and reproduced in other prospective trials [2,3,4]. While SABR has become increasingly utilized over conventionally fractionated radiotherapy (CFRT)-defined here as 1.8Gy-2.5Gy per fraction-for early NSCLC, limited randomized evidence exists to support overall survival (OS), LC advantages, or toxicity benefits [1,5].

Rigorous evaluation of the safety and efficacy of new treatments through randomized trials is paramount to limiting biases found in other study designs [6]. In radiation oncology, widespread adoption of new technologies and techniques based on pre-clinical promise or lower-level evidence have occasionally preceded the development of level 1 evidence, as seen in the adoption of proton beam therapy for adult cancers [7]. While this can increase access to promising treatments, comparative benefits and risks remain unknown. Given SABR’s ubiquity of application with limited randomized data, we conducted a systematic review and meta-analysis to investigate the efficacy and toxicities of SABR compared to CFRT based on randomized comparisons.

## Review

Methods

Search, and Screening of Abstracts

**Figure 1:**
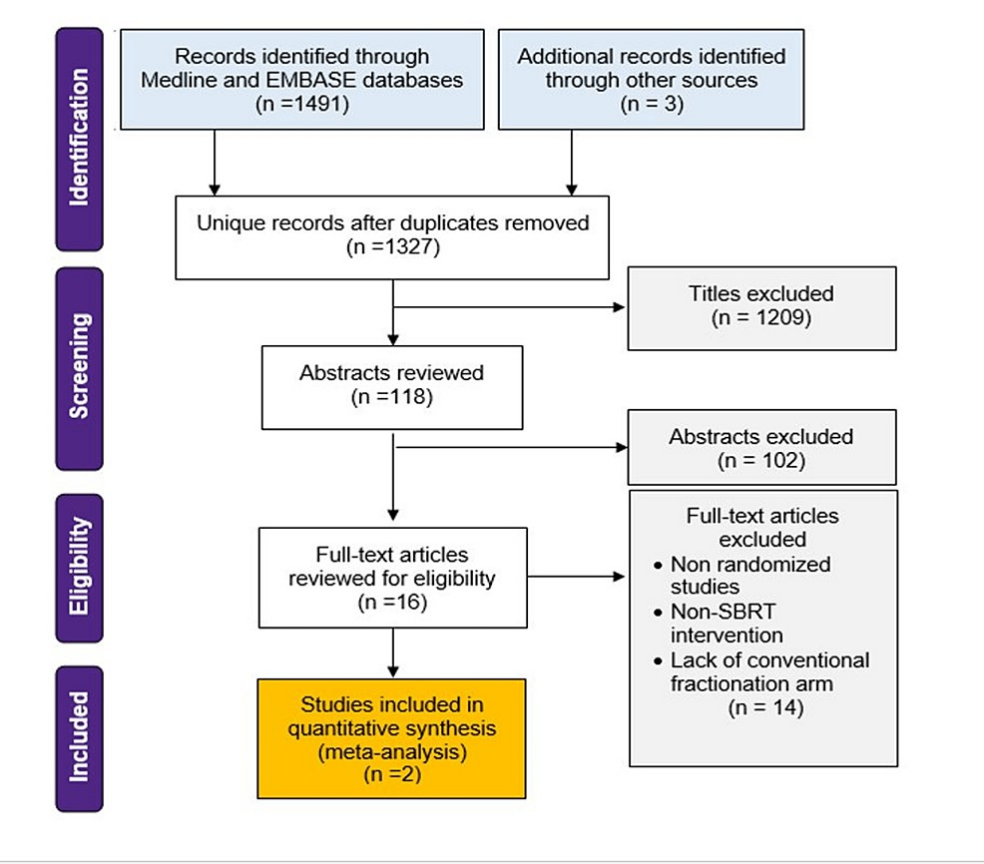
PRISMA Flow Diagram SBRT: Stereotactic body radiation therapy

A systematic review of databases was conducted to identify trials of early NSCLC patients randomized to SABR versus CFRT. Medline, Embase, and Cochrane databases were queried from inception to December 2020. Included trials were randomized phase-II or III trials comparing CFRT with SABR (highly conformal, image-guided, hypo-fractionated) for early NSCLC (T1-T2a, N0) biopsy-proven or highly suspicious lung lesions. Studies were screened by title and abstract, with subsequent full-text reviews by two independent reviewers and a third author resolving discrepancies. Patient and treatment characteristics, and outcome data (LC, PFS, OS, and acute and late toxicity rates) were abstracted by two independent reviewers into an Excel (Microsoft Corp., Redmond, WA, USA) template. Citations from full-text-reviewed manuscripts were examined to ensure inclusivity. A PRISMA diagram is provided in Figure 1. Quality and risk of bias were assessed with the Cochrane risk-of-bias 2.0 (2016) by independent reviewers [8].

Statistical Analysis

Effect estimates including hazard ratios (HR) and confidence intervals (CI) were abstracted, and both fixed and random-effects models were used to estimate treatment effects. Heterogeneity was assessed with tau-squared statistics. Comprehensive Meta-Analysis software version 2 (Englewood, NJ, USA) was used in this analysis. Descriptive statistics were generated and compared by Fisher’s exact test. Commonly reported toxicities of ‘any’ grade, and grade 3 or higher were compared by the Cochran-Mantel-Haenszel test, stratified by trial.

Individual patient data were approximated using DigitizeIt ™ (Braunschweig, Germany). Point estimates were extracted from Kaplan-Meier (KM) survival curve images in both trials as per the methods published by Guyot et al. in 2012 [9]. Survival curves were reconstructed from extracted data and validated against respective KM curves to ensure accuracy. Data were pooled and new KM survival curves were created. A Cox proportional hazard model was used for a pooled HR based on approximated individual patient data. The R software version 4.0.3 (R Foundation for Statistical Computing, Vienna, Austria.) and SAS v9.4 (SAS Institute, Inc., Cary, NC, USA) were utilized for statistical analysis.

Results

Population

The literature search identified 1494 studies, and after the exclusion of duplicates, 1327 were screened. Studies were excluded after the title screen (n=1209) and abstract review (n=102); 16 studies were included for full-text review (Figure 1). Two randomized trials met inclusion criteria, including a total of 203 patients (Table 1), of which 115 (57%) received SABR, and 88 (43%) received conventionally fractionated radiotherapy (CFRT). Characteristics are summarized in Table 2. The mean age was 74, with 48% male. The mediastinal staging was not required. Most patients had T1 cancers (67% T1 vs. 33% T2). Of those with T2 cancers, 64% received SABR and 36% received CFRT. Most patients were Eastern Cooperative Oncology Group (ECOG)-1 (65%), 22% were ECOG-0, and 12% were ECOG-2. Stereotactic ablative radiotherapy was prescribed as 45Gy/3 fractions (at the periphery of planning target volume), 54Gy/3 fractions, or 48Gy/4 fractions. Conventionally fractionated radiotherapy was prescribed as 70Gy/35 fractions, 66Gy/33 fractions, or 50Gy/20 fractions.

**Table 1:** Characteristics of trials included in meta-analysis SABR: Stereotactic ablative body radiation, CFRT: Conventionally fractionated radiation therapy, WHO: World Health Organization, ECOG: Eastern Cooperative Oncology Group, TROG: Trans-Tasman Radiation Oncology Group, MDT: Multidisciplinary team, 4DCT: 4-dimensional computed tomography, GTV: Gross tumour volume, CTV: Clinical target volume, ITV: Internal target volume, PTV: Planning target volume, CBCT: Cone beam computed tomography, Sup-Inf: Superior and inferior dimensions, PET: Positron emission tomography, #: fractions

	Stereotactic precision and conventional radiotherapy evaluation (SPACE) [10]	CHISEL [11]
Author	Nyman et al., 2016	Ball et al., 2019
Number (n)	102 (49 SABR, 53 CFRT)	101 (66 SABR, 35 CFRT)
Enrollment	2007-2011	2009-2015
Design	Randomized phase-II, multi-center	Randomized phase III, multi-center
Primary endpoint	Progression-free survival	Local treatment failure
Randomization	1:1	2:1 (SABR:CFRT)
Key inclusion/ exclusion criteria	WHO 0-2; tumour <6cm; Could not be “adjacent” to trachea, bronchus, esophagus; no pulmonary function test limits	ECOG 0-1; >1cm from mediastinum; >2cm from lobar bronchi; MDT assessed medically inoperable; pulmonary function not specified
Diagnosis	T1-T2a, biopsy/ serial CT growth	T1-T2a, biopsy proven, PET avid
Motion management	Vacuum pillow; GTV + margin for CTV; PTV= CTV+ 5mm and 10mm Sup-inf; 4DCT and CBCT not standard	Immobilization device not specified; ITV=GTV on each phase +5mm PTV=ITV+ 10mm; 4DCT standard; daily CBCT or fiducial insertion for all; pre-trial site visit to validate dose and procedure
Accepted SABR doses	66Gy/3# (45Gy/3# at periphery of PTV)	54Gy/3# or 48Gy/4#
Accepted CFRT doses	70Gy/35#	66Gy/33# or 50Gy/20#
PET Staged (%)	64.7%	100%

**Table 2:** Characteristics of pooled patients Pooled patient characteristics from SPACE [10] and CHISEL [11] trials. *Histology excludes non-biopsied tumours: n=165 of total n=203 SABR: Stereotactic ablative body radiation, CFRT: Conventionally fractionated radiation therapy, Adeno: Adenocarcinoma,  ECOG: Eastern Cooperative Oncology Group, PET/CT: Positron emission tomography/computed tomography

Variable		SABR	CFRT	p-value
Sex	Male (n, %)	58 (50%)	39 (44%)	p=0.40
	Female	57 (50%)	49 (56%)	
ECOG	0-1	103 (90%)	73 (83%)	p=0.21
T Stage	1	73 (63%)	64 (73%)	p=0.18
	2	42 (37%)	24 (27%)	
Biopsy	Completed	96 (83%)	69 (78%)	p=0.37
Histology*	Adeno	48 (50%)	31 (45%)	p=0.53
	Other	48 (50%)	38 (55%)	
PET/CT staged	Completed	96 (83%)	71 (81%)	p=0.71
Invasive mediastinal staging	Completed	Not specified	Not specified	N/A
Total		115	88	

Survival and Control

**Figure 2:**
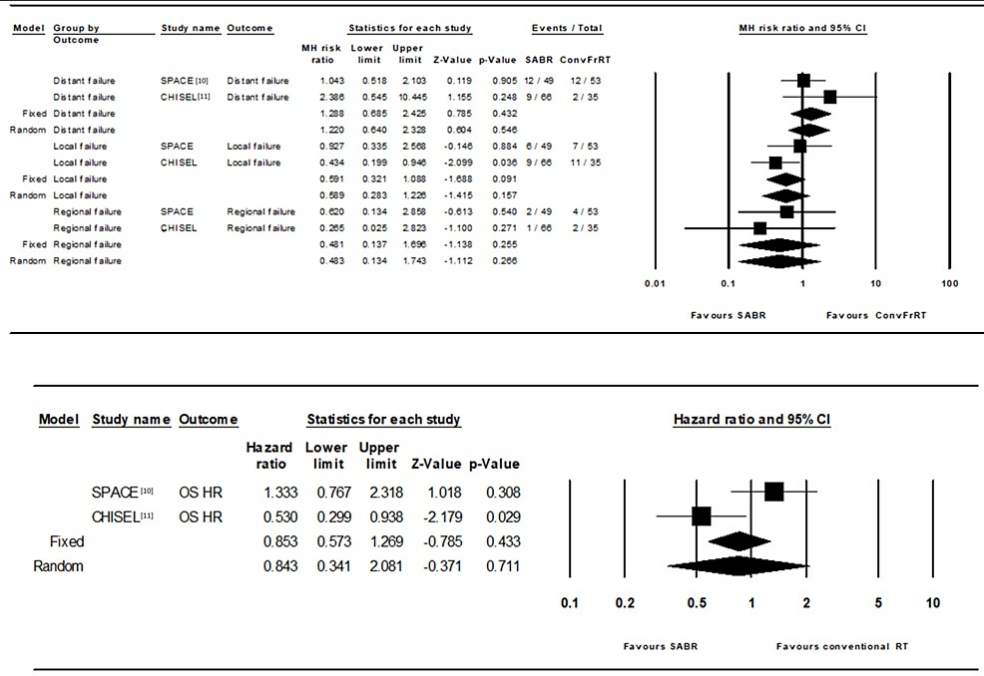
Meta-analysis forest plots for local, regional, distant control, and overall survival CI: Confidence interval, OS: Overall Survival, HR: Hazard Ratio, SABR: Stereotactic ablative body radiation, ConvFrRT: Conventionally fractionated radiation therapy, SPACE: Stereotactic precision and conventional radiotherapy evaluation, MH: Mantel-Haenszel

Stereotactic ablative radiotherapy was not associated with significantly improved OS based on the random effect model from the meta-analysis (hazard ratio: 0.84; 95% CI 0.34-2.08, p=0.71) as shown in Figure 2. Additionally, there was no significant difference in local failures between SABR and CFRT (relative risk: 0.59; CI 0.28-1.23, p=0.16).

**Figure 3:**
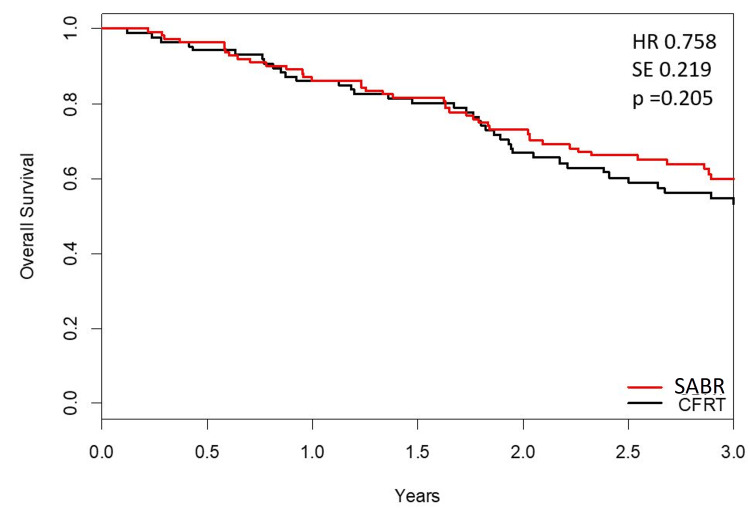
Estimated individual patient data, reconstructed combined Kaplan-Meier curves from SPACE and CHISEL trials HR: Hazard ratio, SE: Standard error, SABR: Stereotactic ablative body radiation, CFRT: Conventionally fractionated radiation therapy, SPACE: Stereotactic precision and conventional radiotherapy evaluation

Figure 3 displays pooled OS KM curve. The median OS for the CFRT arm was 3.12 years (95% CI 2.41) and unreached for the SABR arm (log-rank test p=0.20). The pooled HR obtained from Cox proportional hazard model was 0.758 (p=0.20).

Radiation Toxicity

No difference was identified for severe (grade 3+) toxicity, while one grade 4 toxicity of dyspnea was reported for SABR. There were no grade 5 toxicities. Common adverse events of cough, rib fracture, fibrosis, and pneumonitis were similar between SABR and CFRT. Stereotactic ablative radiotherapy demonstrated less ‘any grade’ toxicity for esophagitis (p=0.0125), dyspnea (p=0.0285), and skin reaction (p=0.0005). Pooled toxicity data is provided below in Table 3.

**Table 3:** Pooled toxicity outcomes Pooled patient characteristics from SPACE [10] and CHISEL [11] trials. * Cochran-Mantel-Haenszel test stratified by trial SABR: Stereotactic ablative body radiation, CFRT: Conventionally fractionated radiation therapy, SPACE: Stereotactic precision and conventional radiotherapy evaluation

Toxicity (any grade)	SABR	CFRT	p-value
Esophagitis	5 (4)	16 (19)	0.013
Pneumonitis	19 (17)	20 (23)	0.581
Dyspnea	56 (49)	61 (71)	0.029
Fibrosis	46 (40)	36 (42)	0.654
Cough	60 (52)	49 (57)	0.837
Skin reaction	22 (19)	39 (45)	<0.001
Rib fractures	13 (11)	7 (8)	0.244
Toxicity (grade 3+)	SABR	CFRT	p-value
Esophagitis	0 (0)	0 (0)	n/a
Pneumonitis	0 (0)	1 (1)	0.341
Dyspnea	7 (6)	5 (6)	0.568
Fibrosis	0 (0)	1 (1)	0.341
Cough	3 (3)	0 (0)	0.145
Skin reaction	1 (1)	0 (0)	0.293
Rib fractures	0 (0)	0 (0)	n/a
‘Any grade 3+’	11	7	0.422

Risk of Bias

Both studies were of low risk of bias in all domains, except for scoring ‘some concerns’ in potential bias from outcomes measurement, and for lack of blinding.

Discussion

Our systematic review identified two randomized studies comparing CFRT with SABR in early NSCLC. Meta-analysis did not demonstrate a significant benefit of SABR for early NSCLC in OS, LC, or toxicity. Similarly, the analysis based on approximated individual patient data did not demonstrate a significant difference in OS. These findings fail to offer conclusive evidence to bolster extensive single-arm study data supporting SABR’s relative benefit in LC, and safety [2,3,9].

The Scandinavian randomized phase-II stereotactic precision and conventional radiotherapy evaluation (SPACE) trial demonstrated no overall survival (OS) or progression-free survival (PFS) benefit at 37 months median follow-up [10]. However, the SABR group had significantly less toxicity, notably less pneumonitis and esophagitis, and higher health-related quality of life (HQROL). In contrast, the CHISEL trial showed OS and PFS advantages in the SABR arm, with no significant difference in toxicity or HRQOL [11].

The findings of this small analysis differ from many single-arm studies with respect to OS. A recent systematic review and meta-analysis of 87 SABR and 24 CFRT curative intent, prospective and retrospective studies for NSCLC revealed significant OS benefits in SABR studies, despite consistently older patient populations [12]. Further, radiobiologic principles are superior with SABR [13], and population-level analysis has also shown the benefit of SABR [14]. There are some possible interpretations of our findings: 1) The analysis is underpowered, and a greater number of large, contemporary randomized clinical trials (RCTs) would likely demonstrate a benefit for SABR. 2) The CFRT performs better than prior retrospective comparisons suggested, making the perceived benefit of SABR overstated [12]. While SABR OS in our analysis was similar to prior meta-analyses, the CFRT cohort outcomes were better than previously reported [12]. It is possible that improvements in radiation simulation and delivery have improved the contemporary outcomes of CFRT. 3) Follow-up is too short, and the relative benefits of SABR may not yet be realized. This is less likely; while greater benefits may be expected over time, differences at two and three years have been noted in retrospective studies [12]. 4) Subtle, non-statistically significant differences between groups obscure the benefits of SABR (e.g., more T2 and biopsy-proven tumours in the SABR arm). Size or T-stage is an established predictor for failure and survival [15], while radiation of potentially pre-cancerous or benign lesions is likely to show improved outcomes.

Examined toxicities revealed no significant differences in grade 3 or higher pneumonitis, esophagitis, or all grade 3+ toxicities. However, we did identify significantly fewer ‘any grade’ toxicities of esophagitis in keeping with the trend of reduced mediastinal toxicities observed in other series [12]. Importantly, ‘any grade’ dyspnea and skin reaction was also better in the SABR arm. Differences in toxicity classification may have been present across trials, as despite similar doses and delivery techniques only one event of esophagitis (grade 1) was noted on CHISEL across both arms, while higher rates were observed in SPACE [10,11]. 

Local control improvement in the SABR arm did not reach statistical significance in this analysis. While all SABR patients received a biologically effective dose (BED) of >100Gy10 in keeping with modern guidelines [16], BED in the positive CHISEL trial was higher, and other factors may contribute to the lack of observed benefit. The non-significantly higher proportion of larger tumors in the SABR group (37% T2 vs 27%, p=0.18), heterogenous access to modern image guidance in one trial [10], and small differences in biopsy rate may have obscured the benefits of SABR. These doses corresponded with LC above 90% at two years in multiple prospective trials [2]. However, a single-arm phase-II study showed a pathologic complete response rate of only 60% at 10 weeks post-SABR [17]. In contrast, a systematic review of studies of CFRT in inoperable patients showed local failure rates ranging from 6% to 70% at two years [18]. Conventionally fractionated radiotherapy control and survival outcomes in analyzed studies were better than many historical references, potentially due to improved staging and contemporary treatment delivery.

In situations of equipoise, alternative outcomes can be useful in determining the standard of care. Stereotactic ablative radiotherapy appears to preserve HRQOL in a systematic review [19]. In a cost-benefit simulation model of a public-payer-funded health system, SABR was projected to be the most cost-effective treatment for inoperable and borderline-operable early NSCLC, dominating CFRT, sub-lobar resection, and best supportive care [20].

The findings of this study should be considered in the context of its limitations. The small number of trials available for meta-analysis is the most prominent limitation, and to our knowledge, no similar trials are accruing, or awaiting analysis. Trial sizes were well balanced, however, one trial randomized at a 2:1, SABR to CFRT, compared with 1:1 in the other. This led to a greater weight of SABR patients from CHISEL and CFRT from the other. The total patient sample size remains small. Variations in exclusion criteria, including the need for biopsy, fluorodeoxyglucose (FDG)-PET scan, use of motion management during treatment, and performance status and lung function, potentially affect results. While the Q and I² value was low, heterogeneity bias cannot be excluded. Despite these limitations, the current study provides meaningful addition to the literature by systematically reviewing the literature to ensure applicable studies are considered. Further, meta-analysis increases the power and effect precision over individual trials. While individual patient data was not obtained, utilizing DigitizeIt to analyze KM curves as an approximation has been shown to be reproducible and accurate, and helps to address limitations of secondary analyses using HRs alone [8]. As further direct comparisons between SABR and CFRT are unlikely given the lack of equipoise in the community, this study represents the best available evidence. Our analyzed outcomes were comparable between modalities. In the context of substantial single-arm, retrospective, biologic, population-based, and surrogate endpoint data in support of SABR, we advocate for it as the standard of care in jurisdictions where the technology and expertise to deliver it safely and effectively exists. Further, factors including reduced visits to the treatment facility make SABR more convenient, accessible, and less financially toxic for patients.

Ongoing trials are investigating the optimization of SABR for efficacy and safety, including safety in ultra-central lesions [21], control for larger lesions [22], and the role of systemic therapy, including concurrent or adjuvant immunotherapy [23]. Hypo-fractionated radiotherapy has been explored as an intermediate option between SABR and CFRT with evidence of good LC and has proved useful in ultra-central tumors where SABR toxicity has been concerning [24]. While a recent propensity-matched analysis of SABR compared with 60Gy/15 fractions demonstrated improvement in both LC and OS favoring SABR [25], results of the phase-III LUSTRE trial are awaited [26]. 

## Conclusions

This systematic review and meta-analysis of randomized trials fail to confirm improvements in LC or OS of SABR over CFRT in early NSCLC despite widespread adoption and extensive single-arm prospective, population, and retrospective studies suggesting its benefit. Toxicity profiles and events were similar. The small sample is likely underpowered to detect clinically significant differences. Given the multitude of non-randomized clinical outcomes and the benefit of non-clinical endpoints, SABR should continue to be considered the standard of care over CFRT in this population.
